# Shortening the Frommelt Attitude Toward the Care Of the Dying Scale (FATCOD-B): a Brief 9-Item Version for Medical Education and Practice

**DOI:** 10.1007/s13187-021-02020-3

**Published:** 2021-05-27

**Authors:** Giorgia Molinengo, Barbara Loera, Marco Miniotti, Paolo Leombruni

**Affiliations:** 1 Department of Psychology, University of Turin, Turin, Italy; 2 ‘Rita Levi Montalcini’ Department of Neuroscience, University of Turin, Turin, Italy; 3 Clinical Psychology Unit, AOU ‘Città Della Salute e della Scienza’, Turin, Italy

**Keywords:** FATCOD, Attitudes, Medical students, Medical education, Palliative care, Assessment

## Abstract

End-of-life care training has gaps in helping students to develop attitudes toward caring for the dying. Valid and reliable assessment tools are essential in building effective educational programmes. The Frommelt Attitude Toward the Care Of the Dying scale (FATCOD-B) is widely used to measure the level of comfort/discomfort in caring for the dying and to test the effectiveness of end-of-life care training. However, its psychometric properties have been questioned and different proposals for refinement and shortening have been put forward. The aim of this study is to get to a definitive reduction of the FATCOD-B through a valid and parsimonious synthesis of the previous attempts at scale revision. Data were gathered from a sample of 220 medical students. The item response theory approach was used in this study. Of the 14 items selected from two previous proposals for scale revision, 3 had a weak correlation with the whole scale and were deleted. The resulting 11-item version had good fit indices and withstood a more general and parsimonious specification (rating scale model). This solution was further shortened to 9 items by deleting 2 of 3 items at the same level of difficulty. The final 9-item version was invariant for gender, level of religiosity and amount of experience with dying persons, free from redundant items and able to scale and discriminate the respondents.

## Introduction

The relationship between healthcare workers and patients influences the outcome of any treatment intervention, both for medical and nursing care. The care of terminally ill patients often involves the requirement to respond to a broad spectrum of needs, to face not only physical suffering but also psychological and existential distress and to deal with the finitude of medical science and thus with frustration in terms of separation and loss [1, 2]. Developing an effective caring relationship with a terminally ill patient is a tall order and requires personal skill and the correct attitude [3, 4]. Accordingly, there has been an increase in palliative care curricula in medical and nursing schools in recent decades worldwide. Medical educators have defined guidelines on essential contents for adequate training in palliative care and proposed teaching methods to train medical and nursing students on interpersonal and communication skills in end-of-life care, often from a teaching perspective [5–8].

However, less attention is paid to the learning perspective, with the result that the process of psychological growth and development of attitudes toward the care of dying patients is almost neglected [3, 9]. This is a major shortcoming in nursing but mostly in medical training. This is evidenced by the fact that many medical students and newly qualified doctors feel inadequately prepared, by their studies, for dealing with dying patients [10–12].

To address this gap in medical education, it is important first to assess the students’ background level of personal disposition toward the care of the dying and then to measure changes in attitudes in response to training. Few tools have been developed thus far for evaluating medical and/or nursing students’ attitudes toward caring for the dying. However, there is the Frommelt Attitude Toward the Care Of the Dying scale (FATCOD), which was specifically developed to test the effectiveness of interventions in end-of-life care training and provides a single measurement of the level of comfort/discomfort related to caring for the dying patient and family members [13]. The scale was originally developed for nurses but, in its form for health profession students (FATCOD-B), it has been used with nursing and medical students in numerous studies and is currently available in multiple languages [14]. The authors of this study have contributed to the validation process of the Italian version of the FATCOD-B and proposed an initial refinement of the scale, having ascertained some malfunctioning in the wording of the items [15, 16]. The authors investigated the scale dimensionality quite thoroughly, as, although FATCOD was designed as a single-dimension measure, previous studies yielded conflicting results in this regard [15]. In a recent study, the authors, using an approach based on the confirmatory factor analysis (CFA) and Rasch model, provided clear evidence about the two-dimensional construct of the scale and pointed out that one dimension alone has appreciable measurement and discriminant capabilities so as to be considered a reliable measure for use in palliative care training in medical curricula [16]. More recently, pooling data from nurses and nursing students gathered in previous studies, Swedish authors firstly proposed a shortened version of the FATCOD scale, suggesting retaining 9 of the original 30 items [17]. It is the opinion of the authors that it would be interesting to obtain further statistical evidence on the actual convenience of shortening the scale and the adequate application of the new shortened version among students originating from different educational programmes.

The aim of this study is to propose a definitive reduction of the FATCOD scale by joining together both the Swedish and the Italian contributions [15–18], as, with the different statistical approaches used and the different samples considered, they yielded similar results. With the aim of preserving the usefulness of the tool and facilitating its application in end-of-life care training, this study offers a path toward a valid and parsimonious synthesis of the previous attempts at scale reduction.

## Methods

### Procedures

Item response theory (IRT) was applied in this study. The IRT defines the connections between elements, responders and latent constructs. Imagining the latent construct measured on a continuum, the IRT positions both the items and the individuals along it using the same unit of measurement (logit). To date, two proposals for FATCOD scale revision have been put forward: the first proposed by the authors of this study based on a sample of second-year medical students [16] and the second proposed by Browall and colleagues based on a sample of nurses and nursing students [17]. Bearing in mind the sample differences between the two studies, the empirical evidence needed to combine the previous proposals for the revision of the FATCOD and its further reduction was obtained through a logical path including the following steps:

1. Testing the mixed set of 14 items resulting from the two independent revisions;
2. Reducing, if possible, this set to a non-redundant solution, in terms of item difficulties and/or discriminant power;
3. Testing the robustness of the more parsimonious solution, i.e. the solution with the smaller number of items and the more general model specification (rating scale model instead of partial credit model);
4. Producing evidence of scale invariance across relevant groups (defined by gender, religiosity level and psychological wellbeing);
5. Assessing the factual use of the item answer scale.

### Participants

The participants in the study were 220 s-year Italian students from the Medical School at the University of Turin. They completed the paper-and-pencil questionnaire at the beginning of a course on the doctor-patient relationship developed by the University of Turin Medical School for second-year students. Their average age was 21 years (Sd = 2.4); 57.7% were female. The participants were fully informed of the purposes and methods of the study and voluntarily agreed to participate. The study meets the principles of the Declaration of Helsinki and obtained ethical approval from the Ethical Review Committee at the University of Turin (protocol no. 190231, 5 July 2014).

### Measure

The FATCOD-B scale is a self-administered 30-item questionnaire scored on a 5-point Likert-type scale measuring attitudes toward caring for dying patients and family members [14]. The Italian translation of the FATCOD-B was defined by Mastroianni and colleagues [19]; the psychometric properties of the scale were established by the authors among medical students [15] and by Mastroianni and colleagues among nursing students [20]. A refined version of the scale previously defined by the authors [16] was used in this study.

### Statistical Analyses

To assess the psychometric properties of the FATCOD scale, the Rasch model was estimated using a joint maximum likelihood method. Two types of Rasch models can be chosen to analyse polytomous items: the rating scale model (RSM) [21] and the partial credit model (PCM) [22]. The RSM constrains all thresholds of responses to be distributed identically across all items, while the PCM does not establish constraints on the thresholds. The Rasch model assumes unidimensionality and it was tested by the post hoc principal component analysis of residuals. The critical value ≤ 2 for the eigenvalue was chosen as the rule of thumb in the identification of a second dimension [23]. The INFIT and OUTFIT mean square statistics were used to investigate the degree of misfit of each item and the ideal values for both were about 1.0, with the 0.5–1.5 range considered satisfactory. INFIT is sensitive to unexpected response of persons with an ‘ability’ level near to the item difficulty, while OUTFIT is sensitive to unexpected response observations distant from the item difficulty level [24]. The Point-Measure correlation (i.e. a measure of the correlation between single-item scores and the Rasch measure) was reported and values ≥ 0.30 were considered acceptable. We also considered the person separation index (PSI), which can be used to establish the number of statistically distinct levels—[(4PSI + 1)/3]—of a person’s ability that the items have distinguished. Moreover, category fit statistics (i.e. threshold), as well as category probability curves, were used as diagnostic tools. Differential item functioning analysis (DIF) was performed to test measurement invariance; a difference of at least 0.5 logit between groups indicates an item bias [25]. Statistical analyses were performed on WINSTEPS 3.72.3 (Beaverton, Oregon).

## Results

This study examined two revisions of the original 30-item FATCOD in which the scale was reduced to a subset of functioning items [16, 17]. Seven items (8, 9, 11, 13, 14, 26, 28) were selected in both studies. Five items (3, 5, 6, 7, 15) were only retained in the previous study of the authors [16] and 2 items (25, 29) were isolated in the study of Browall and colleagues [17]. Considering the results of these two independent revisions, a joint set of 14 items was tested using IRT models (partial credit model (PCM) and rating scale model (RSM) specifications).

The results indicate that only PCM specification adequately fitted the data, as the post hoc principal component analysis of the residuals yielded a value of 2, while RSM specification showed a violation of the assumption of one dimensionality, with the first eigenvalues of the principal components analysis equal to 2.1.

Table 1 presents the items in misfit order: 3 items (25, 29, 28) deviated from the model expectations and showed a point-measure correlation value ≤ 0.30 and they were therefore deleted from the subsequent analysis.

**Table 1:** FATCOD: items misfit order, location and fit statistics (14 items, partial credit model specification)

ITEM	LOCATION Logits	INFIT MNSQ	OUTFIT MNSQ	PT-Measure correlation
25	− .90	1.50	1.55	.17
29	− .11	1.28	1.26	.18
28	.99	1.14	1.25	.26
11	.66	1.07	1.10	.38
8	− 1.10	1.10	1.09	.46
9	− .15	1.07	1.07	.42
7	.49	.95	.94	.53
5	.17	.89	.93	.58
13	.52	.91	.90	.54
14	− .21	.87	.85	.62
15	.66	.86	.85	.59
26	− .40	.81	.78	.65
3	− .71	.79	.78	.67
6	.08	.75	.74	.68

The 11-item version displayed one dimensionality, with the first eigenvalue of the principal components analysis equal to 1.8, for both the PCM and RSM specifications. The RSM specification was preferred as it assumes the same structure of the rating scale for all items, thus being more economic than the PCM specification in terms of estimated parameters.

The INFIT (min = 0.77, max = 1.24) and OUTFIT (min = 0.76, max = 1.27) statistics were satisfactory for all 11 items. The item locations ranged from − 1.30 to + 1.09 logit and all 11 items had ordered threshold. The PSI was 1.91 and this indicates that the instrument identifies approximately three (2.88) statistically distinct strata of attitude toward the care of the dying.

**Fig. 1:**
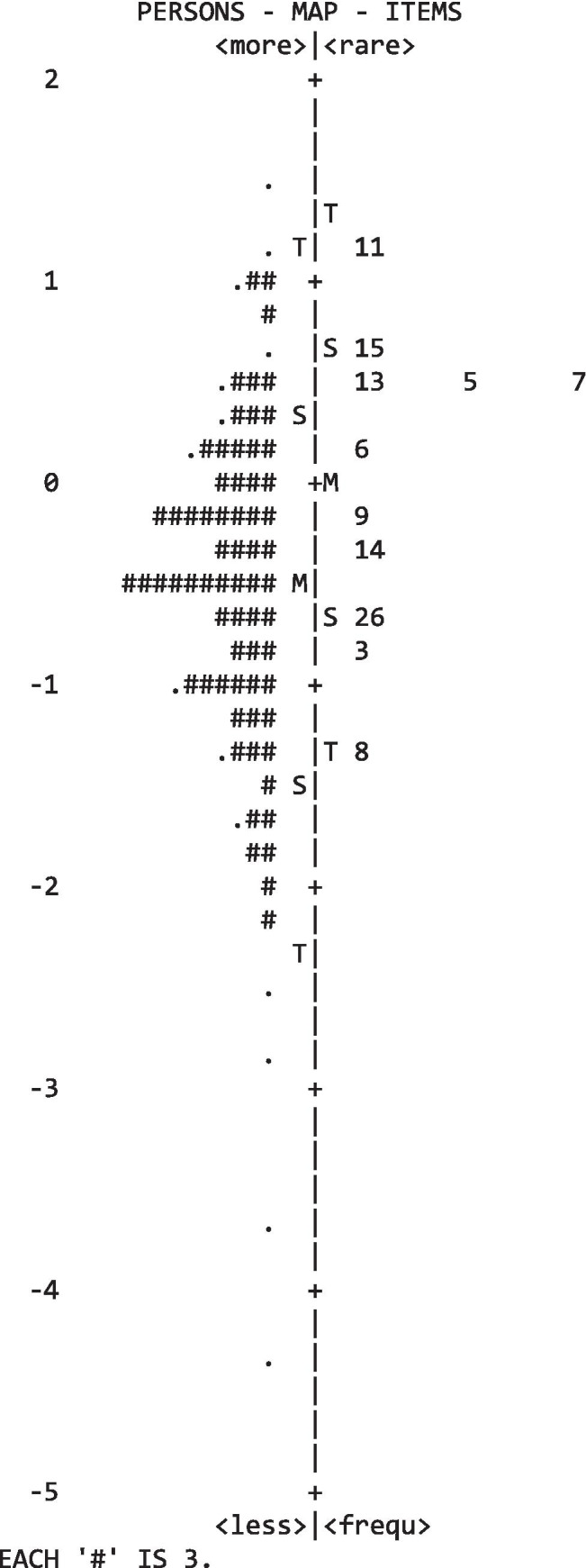
Item person map considering 11 items

An inspection of Fig. 1 revealed that the items were properly distributed along the scale in terms of ‘difficulty’, with items covering both the lower and higher extremes of the continuum of the responder’s level of attitude toward the care of the dying.

Moreover, the 11-item shortening was found to be invariant across discriminating groups: DIF analysis showed that there was no differential item functioning between gender, religiosity and psychological wellbeing. With an observed range of 0.00–0.05, DIF analysis indicated that the 11-item scale works in the same way by contrasting the response function for each item across the different groups.

**Fig. 2:**
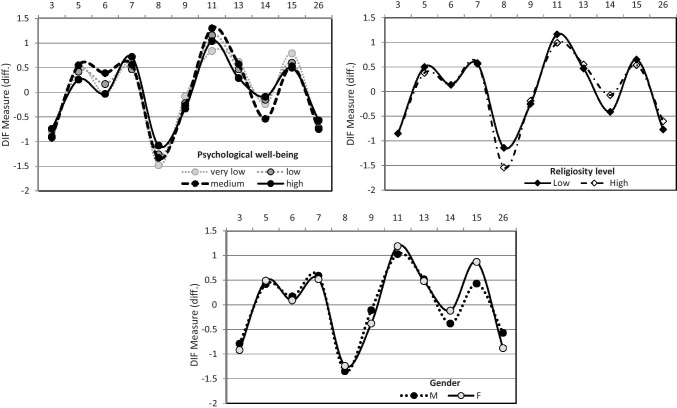
11-item scale invariance across groups: DIF analysis

Ultimately, the map (Fig. 2) revealed that 3 items (13, 5, 7) shared the same level of difficulty. This means that they can be used interchangeably to differentiate the level of attitude of the respondents. Being syntactically the simplest, the authors proposed to retain item 5, suggesting a final FATCOD shortened version composed of 9 items.

## Discussion

This study stems from the need felt by the authors to join together the contributions of two independent research strands aimed at revising the FATCOD scale and ensuring its application in medical and nursing palliative care education [15–18]. The recent study by Browall and colleagues [17] considers a large but heterogeneous sample of in-home care staff (*N* = 1000, including nurses having different professional experience and nursing students), recruited in multiple earlier studies. Conversely, the previous study of the authors included 200 undergraduate medical students, which is a smaller but more homogeneous sample, being all medical students from the same academic year (i.e. the second year) [16]. Due to their differences in knowledge and experience, the two-sample populations probably responded differently to the items. Nevertheless, the two studies revealed comparable performances for the FATCOD scale.

The procedure used by Browall and colleagues to describe the data and to detect malfunctioning items was focused on preliminary analyses based on item distribution, inter-item and item-total correlations [17]. In light of the results from these analyses, the authors decided to delete 20 items and subsequently more sophisticated analyses were performed on the remaining 10 items of the scale and on the same set of cases. Consequently, the measurement of a one-dimensional construct became plausible.

The strategy followed by the authors of this study presented in two previous articles [15, 16] can be considered more conservative. The cues of malfunctioning items were collected by means of different analyses performed on two separate samples of medical students: item analysis, scale reliability and exploratory factor analysis (EFA) on a first sample (*N* = 300), and confirmatory factor analysis (CFA) on a second sample (*N* = 308). The worst items were identified and progressively omitted in modelling the FATCOD scale; testing required specification to ensure that the loss of information caused by item elimination yielded a measurement benefit. This strategy led to a subset of 18 valid items with a two-dimensional structure, with only the first one, consisting of 11 items, considered as a valid, robust and scalable measurement of the sub-construct ‘positive attitudes toward the care of the dying person’ [15, 16].

In summary, the authors of this study insisted on scale dimensionality and tested the structure of the construct underlying the FATCOD scale, adopting the so-called classical test theory (CTT) perspective. Based upon the original theoretical definition, the authors demonstrated that FATCOD has a two-dimensional structure, with only one substantive and psychometrically grounded dimension. Moreover, combining CFA with Rasch models (IRT approach), a subset of 11 valid items (3, 5, 6, 7, 8, 9, 11, 13, 14, 15 and 26) was identified and considered valid to be administered to medical students [15, 16]. Conversely, the Swedish researchers selected a subset of functioning items using predominantly the IRT approach, assuming firstly that the FATCOD scale has a one-dimensional structure and concluding that the tool can be proficiently reduced to 9 items (8, 9, 11, 13, 14, 25, 26, 28 and 29) [17].

**Fig. 3:**
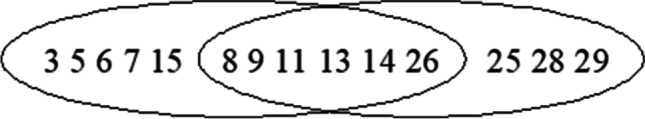
Venn diagram of Italian and Swedish FATCOD reduction

From an inspection of Fig. 3, it is clear that the two solutions proposed contain 6 items in overlap, and 5 + 3 disjointed items. Moreover, all selected items pertain to the same sub-construct or dimension (i.e. positive attitudes toward the care of the dying person) identified by the authors of this study [16], with the exception of item 28. The prominence of the item subset referring to the attitude toward the care of the dying can be considered a robust result in which both approaches converged, in spite of their specificities. Moreover, this result seems to imply that shortening the FATCOD scale means measuring only the first (‘attitudinal’) component identified by the authors previously [16] which corresponds only partly to the original construct proposed by Frommelt, which also included a part related to normative beliefs about dying patients and family members. Thus, the research question is reduced to identify the best item subset able to do this.

To address the question, this study started with the largest set possible, considering the mixed solution emerging from the previous independent solutions. Fit indices of the PCM highlighted that items 25, 28 and 29 had a weak correlation with the scale of 14 items, suggesting that working without these three items could be productive. The 11-item version produced good fit indices and proved to stand up to a more general and parsimonious specification (RSM) which assumes that all answer scales are equal. For the selected item, the answer scale is the same and it also seems to be fully meaningful and useful, as the respondents clearly used and differentiated each ordered category within the proposed answer scale. This solution was further shortened to 9 items by deleting items 7 and 13 which presented the same level of ‘difficulty’ as item 5 (in terms of adherence to the item content and its affective value). Thus, the definitive shortened version was composed of items: 3, 5, 6, 8, 9, 11, 14, 15 and 26.

This study demonstrates that the shortened FATCOD scale proposed here can easily be administered and managed. As the items actually have the same answer scale, a simple sum is a proper score for calculating the level of a positive attitude toward the care of the dying. In addition, the shortened version proposed in this study demonstrated two appreciable properties: (1) the measurement in logit is well scaled and distributed along with a satisfying large range that allows the respondent’s level of attitude as well as the item difficulties to be appropriately discriminated and (2) the scale is stable (i.e. invariant between subgroups generated considering the participants’ gender, level of religiosity and amount of previous experience with dying persons).

### Limitations

This study has limitations that should be considered when interpreting its findings. Firstly, it is not possible to generalise the results observed here for all Italian medical students or for students originating from foreign medical schools. Although the sample size is sufficiently large for the statistical analyses performed, it is nevertheless small and built from a single site. Secondly, this study seeks to integrate its results, observed on a sample of medical students, with those found by Browall and colleagues [17] on a population of nurses and nursing students, and this may be questionable. Further studies on nursing students are required in order to ascertain the validity and reliability of the FATCOD scale solution proposed for medical students in this study and to explore how it works with healthcare workers who have different professional experiences.

## Conclusion

The solution proposed in this study to revise the FATCOD scale consists of a shortened 9-item version with no redundant and equivalent items which, in the opinion of the authors, represents the best heuristic for detecting the attitude that the scale intends to measure. In this version, the scale seems valid and reliable to identify medical students’ attitudes toward the care of the dying and to discriminate students with different levels of attitude regardless of gender, religiosity and amount of previous experience with dying persons. It is also easily administered, completed and scored. The authors of this study suggest testing the strength of the scale they have proposed herein further studies involving different healthcare workers and health profession students and evaluating its sensitivity to change after training interventions or over time.
